# A Service Evaluation of a Specialist Multi‐Disciplinary Weight Management Service Based in Primary Care Including Long‐Term Follow‐Up Data

**DOI:** 10.1111/cob.70096

**Published:** 2026-07-02

**Authors:** Helen M. Parretti, Carly A. Hughes, Sally Erskine, Nicholas Steel, Amy Jennings

**Affiliations:** ^1^ Norwich Medical School University of East Anglia, Norwich Research Park Norwich UK; ^2^ Fakenham Medical Practice, Meditrinia House Norfolk UK; ^3^ School of Biological Sciences Queens University Belfast Belfast UK

**Keywords:** general practice, obesity, primary care, specialist weight management service

## Abstract

This service evaluation of a primary care‐based specialist weight management service reports data from 1094 patients over 5 years (2014–2019), including weight data at 1‐year post‐discharge. The results show clinically and statistically significant improvements in weight, diet, physical activity, quality of life, blood pressure and blood glucose control (in people living with type 2 diabetes). Change in weight was statistically significant for all timepoints in all subgroups. At 1‐year completers (*n* = 560) had lost a mean of 8.3 kg (SD 0.3) and 133 patients (23.8%) had lost ≥ 10% of their starting weight. Using baseline observation carried forward analysis on the whole cohort (*n* = 1094) the mean weight loss at the end of the 1‐year programme was 4.5 kg (SD 0.2) and 144 (13.2%) had lost ≥ 10% of their starting weight. A year after discharge completers demonstrated a mean weight loss of 8.3% (SD 10.3 *n* = 303) and 35.6% (*n* = 108) of completers had maintained ≥ 10% change in body weight. Analysis showed a mean weight loss of 2.5% (SD 6.8 *n* = 1094) in the whole cohort using baseline observation carried forward, demonstrating maintenance of weight loss and suggesting that specialist weight management services in primary care may be effective in the longer‐term.

## Highlights

- What is already known?
  ○ Obesity is a significant and growing health problem, associated with multiple co‐morbidities, decreased life expectancy and increased health costs. Weight loss is associated with improvements in co‐morbidities and reduces premature mortality.
  ○ Systematic reviews have suggested that specialist weight management services can have a short to mid‐range positive effect on body mass index.
  ○ Systematic reviews have indicated that weight management services in primary care can be effective to achieve moderate weight loss, but good quality long‐term data on weight loss maintenance are sparse.
- What this study adds?
  ○ This evaluation analyses 5 years of data (1st April 2014 to 28th March 2019) and includes data from 1094 patients attending a primary care based multidisciplinary specialist weight management service, the Fakenham Weight Management Service.
  ○ Data show consistent results over time, demonstrating clinically and statistically significant improvements in weight, diet, physical activity, quality of life, blood pressure and blood glucose control (in people living with type 2 diabetes).
  ○ Long term weight loss data a year after discharge in completers demonstrated a mean weight loss of 8.3% (SD 10.3 *n* = 303) using all data available. Baseline observation carried forward analysis showed a mean weight loss of 2.5% (SD 6.8 *n* = 1094) a year after discharge in the whole cohort demonstrating maintenance of weight loss and suggesting that specialist weight management services in primary care may be effective in the longer‐term.
  ○ The results confirm the feasibility and effectiveness of providing a specialist weight management service in primary care, which is particularly relevant due to the high prevalence of obesity and with the recent introduction of new pharmacotherapies.

## 1 Introduction

Obesity is a complex condition, with multiple causal factors, requiring a range of approaches from public health policies aimed at prevention to therapeutic interventions focusing on management. Obesity is recognised as one of the most important public health problems currently facing the world, with an estimated 42% of adults living with overweight or obesity worldwide and 0.81 billion adults globally living with obesity in 2020 [1]. The Health Survey for England (HSE) 2022 reported that 29% of adults were living with obesity and 64% were living with overweight/obesity based on BMI criteria [2]. Obesity is associated with a higher risk of co‐morbidities, ill health and premature mortality, as well as increased health and social costs [3, 4, 5, 6]. In the UK, the economic cost to the NHS and wider economy is estimated at £11.4 billion, with lost productivity, unemployment and social care costs of £74.3 billion [7]. Weight loss can improve health and mortality in people living with obesity (PLwO) [5, 6, 7, 8]. Modest weight loss of around 5% has been shown to be clinically beneficial, although a weight loss ≥ 10% is usually required to achieve remission of type 2 diabetes (T2DM) [4]. There is evidence that even with weight regain post‐intervention, weight management interventions may lead to some weight loss being maintained in the longer term [9, 10, 11] and that there is a legacy benefit on cardiovascular health after weight loss even with some weight regain [5, 12].

The UK NHS tiered system classified public health interventions as Tier 1, community group interventions Tier 2, specialist weight management services (SWMS) Tier 3 and bariatric surgery Tier 4 [13]. A freedom of information (FOI) request in England which included replies from all 42 Integrated Care Boards (ICBs (local commissioning bodies)) revealed that access to SWMS was unevenly distributed and inequitable [14, 15]. Only about half of the ICBs (24/42) commissioned both Tier 3 SWMS and Tier 4 bariatric surgery and 15/42 reported problems such as services being closed to new patients (7/42) or covering only part of the population [14, 15]. Four ICBs did not commission any SWMS. Bariatric surgery services were restricted, with inconsistent entry criteria, which did not all reflect National Institute for Health and Care Excellence guidance [16]. An additional FOI based paper reported that 34/41 Integrated Care Systems provide commissioned medical SWMS and 34/41 fund bariatric surgery [17]. Thirteen reported not complying with NICE guidance and there remains significant geographical variation in availability of services [17].

Secondary care‐based SWMS are oversubscribed, with long waiting lists or closed to new referrals [14, 15]. This is despite evidence that only a small percentage of PLwO are referred to publicly funded weight management services [18]. An observational cohort study (using data between 2007–2020) revealed that only 3.13% of people recorded with overweight/obesity were referred to weight management services [18]. If referral rates increased in line with current NICE guidance [16] secondary care services might be overwhelmed. Primary care could be well placed to deliver local specialist services if given the necessary resources. However, there is limited evidence for the effectiveness of SWMS based in primary care [19, 20, 21, 22] and a lack of data on long‐term weight maintenance [23].

This paper provides data that will contribute to the evidence base for primary care‐based specialist weight management services in the United Kingdom including longer‐term weight loss outcomes. It is important to note that GLP‐1 agonist obesity management medications were not available to NHS patients during the service evaluation period. The recent approval of Tirzepatide by NICE, which includes its use outside of hospital settings such as primary care [24] is likely to encourage primary care‐based prescribing supported by behavioural advice. Whilst many people may benefit from remote behavioural support and local prescribing, complex patients may need more specialised medical, psychological or dietetic support.

### 1.1 Description of Service

**FIGURE 1:**
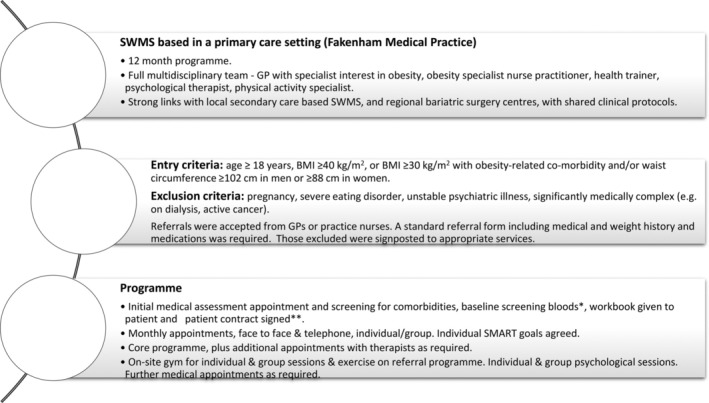
FWMS brief service description.

**FIGURE 2:**
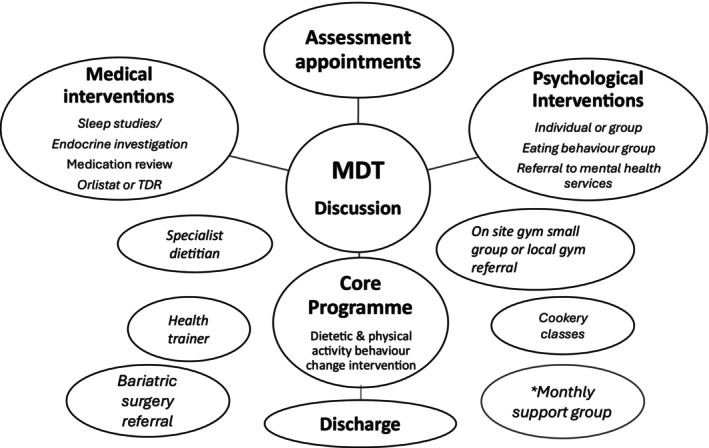
FWMS patient pathway.

The Fakenham Weight Management Service (FWMS) provided specialist multicomponent, multidisciplinary services to people with complex or severe obesity (BMI ≥ 35 kg/m^2^ plus co‐morbidities or BMI > 40 kg/m^2^). Patients with significant medical complexity such as those requiring dialysis were excluded and referred to secondary care services. FWMS was based in Norfolk, UK and set up in 2009 and a full description of the service and results have been previously reported [20]. A brief description of the service and FWMS patient pathway are given in Figures 1 and 2. The service met NICE criteria for a Tier 3 SWMS in place at the time [25, 26]. Commissioning information is available in the Supporting Information. All the clinical staff had appropriate qualifications and in addition had undergone the World Obesity SCOPE accreditation training (further details Supporting Information). FWMS was also recognised as a specialist centre for obesity management by the Association for the Study of Obesity (ASO) [27] and the European Association for the Study of Obesity (EASO) [28].

The primary clinical outcome measures for evaluation of the service were categorical weight loss of 5% or 10% at 12 months in completers. A Key Performance Indicator (KPI) was to achieve at least 5% weight loss in 70% of completers at 12 months. A completer was defined as a person who had attended for 75% of the programme (at least nine visits, usually over 9 months). Additional KPI's are described in the Table S1.

Secondary Outcomes for Evaluation Included

- An improvement in Quality of Life (QoL) scores measured using Euroqual EQ‐5D‐5L VAS (Visual analogue scale) [29]
- An increase in physical activity measured using the General Practice Physical Activity Questionnaire (GPPAQ) [30].
- An increase in daily fruit and vegetable consumption measured by 2 item food frequency score (2 item FFS) fruit and vegetables [31].

The programme involved initial medical assessment by a clinician (GP or Advanced Nurse Practitioner (ANP)). Weight was measured using regularly calibrated specialist digital bariatric scales and wheelchair scales were available. Physicians saw 90% of participants; their role was to assess and manage complex co‐morbidities, review and action screening blood results and to identify undiagnosed obesity‐related co‐morbidities such as type 2 diabetes, depression and obstructive sleep apnoea. Assessment followed the principles described at the time in the Obesity Canada 5As guidance [32] and NICE CG189 [25]. The FWMS GP/ANP communicated with the participants' own GP or the secondary care specialists when appropriate. They would also advise on suitability for NHS pharmacotherapy (Orlistat), Total Diet Replacement (TDR) and bariatric surgery. A medical opinion on fitness to exercise was sometimes required.

The FWMS pathway is described in Figure 1 (more details in Supporting Information). Most contacts were face‐to‐face at Fakenham Medical Practice (a large North Norfolk GP surgery with bariatric equipment, plentiful parking and on‐site pharmacy). However, during COVID‐19 online contacts were provided. In addition to the core programme, all patients interested in bariatric surgery and meeting NICE eligibility criteria [25] were reviewed by the GP/ANP and psychological therapist on at least two occasions. A maximum of six individual psychology appointments per participant were provided and where necessary referrals were made to mental health, psychology or eating disorders services. Many patients struggled with emotional eating, cravings, binge eating, night eating and poor sleep. There is evidence to support the efficacy of psychological interventions within an MDT service [33] and FWMS developed groups to help manage these behaviours. If patients met the criteria for bariatric surgery and the MDT agreed, a detailed report, including a pre‐surgical checklist, was provided for the patient's GP to use for an onwards bariatric surgery referral.

Those identified as clinically appropriate for Total Diet Replacements (TDR) [34, 35], had additional GP/ANP and dietitian appointments and support including medication reviews. The TDR provided either used a milk‐based diet with vitamin supplementation or a commercial TDR. An NHS discount scheme was negotiated with a reputable commercial provider of TDR products (LighterLife).

A post‐discharge patient‐led support group met at monthly intervals with open access for all those who had attended the programme. A further monthly support group for those either awaiting bariatric surgery or post bariatric surgery was provided in response to patient feedback (Figure 2).

## 2 Methods

This was a retrospective service evaluation and only data routinely collected were available for analysis. Demographic data were collected at baseline (entry into the service); this included the Edmonton Obesity Staging System (EOSS) score [36]. This is a five‐stage system of obesity classification that considers medical, psychological and functional parameters according to severity with scores ranging from 0 to 4. The EOSS score has been reported to be a better predictor of mortality than BMI [37]. It was used to compare the complexity of different populations attending SWMS. Weight data were collected monthly in person on calibrated scales and secondary outcome data were collected quarterly at appointments and recorded in the clinical records. A limited number of weights were reported from GP records in those patients who dropped out of the programme. Data were entered on a master spreadsheet. There was a technical problem with the master spreadsheet at the midterm point, relating to NHS software updates, which resulted in some newly entered weights not being saved. This was picked up after 1 month when the next weights were entered and at that point the missing weights were re‐entered on the spreadsheet from the medical records. It is possible that a small number of weights may have remained missing.

Core outcome data were recorded on a Microsoft Excel spreadsheet. Reliable data were recorded for the EQUATION 5D‐5L Visual analogue scale (VAS) on the master spreadsheet, but the other EQUATION 5D‐5L domains were recorded using different software and we were unable to access those data for the purposes of this evaluation. Additional data such as discharge blood pressure were less reliably documented. All those referred had a baseline HbA1c measured as part of entry criteria and for some participants with type 2 diabetes mellitus (T2DM) HbA1c was available from their GP at 12 months. Further anonymised demographic data on number of referrals per annum, recruitment and subsequent attendees and additional data derived from the annual reports supplied to commissioners are included in the Supporting Information (Tables S2 and S3).

The one‐year post discharge follow‐up data included data from: 12 months FWMS follow‐up face‐to‐face appointments offered to all completers, data from GP records, weights taken at the FWMS long‐term support group and completers' self‐reported weights submitted in response to mail requests. The majority of follow‐up weights were recorded in person at FWMS or from GP records. There was no funding to contact participants who had dropped out during the programme, but some of their weights were available from GP records.

The cohort reported on (all starters) included patients who undertook meal replacement diets, those on Orlistat and those preparing for bariatric surgery. The analysis involved the whole cohort and did not differentiate between those groups.

### 2.1 Statistical Analysis

The raw data were checked and cleaned and anonymised by C.A.H. Any duplicate data were removed by AJ. AJ performed the statistical analysis.

Continuous values in the text and tables are presented as mean (standard deviation; SD) and categorical data as percentage (*n*), unless otherwise stated. Baseline characteristics of completers and non‐completers of the programme were compared using independent sample *t*‐tests or Pearson *χ* ^2^ tests with a completer defined as having attended nine appointments and having a weight recorded at 12 months. Differences between pre‐ and post‐programme measurements were compared by paired sample *t*‐tests. Data were analysed on an intention‐to‐treat like basis, with the value at the previous assessment carried forward if a value at the relevant timepoint was not available (both last observation carried forward (LOCF) and baseline observation carried forward (BOCF) analyses were conducted).

Regression analyses were used to assess predictors of weight change with change in weight in kg as the dependent variable and the relevant predictors as the independent variable; in model 1 each predictor was modelled individually and for model 2 all predictors were entered into the same model. *p*‐values < 0.05 were considered statistically significant (with Bonferroni‐adjusted statistical significance also indicated). Statistical analyses were performed using Stata software (version 18; College Station, TX, USA: StataCorp LLC).

The study was approved as a service evaluation by the UEA ethics committee: reference number ETH2223‐123 (12/01/2023).

## 3 Results

Total referrals made to the service over the 5‐year period were 2010, with 1094 participants attending the service. A summary of the total referrals and subsequent pathways is included in the Supporting Information (Table S2), annual referrals are presented in Table S3.

Baseline characteristics of the FWMS patients are shown in Table 1. Ethnicity of FWMS patients was < 1% non‐White, compared with NHS Norfolk and Waveney Clinical Commissioning Group (NWCCG) 3.3% and England 14.6% (2020) [38]. The age distribution of FWMS patients was 16–24 years 4.3% (NWCCG 9.7%, England 10.5% 2020), 25–64 years 82.3% (NWCCG 48.5%, England 51.8% 2020) and 65 years and over 13.4% (NWCCG 25%, England 18.5% 2020) [38].

**TABLE 1:** Baseline characteristics.

Characteristic	*n*	Mean (SD) or *n* = (%)
Age, years	1092	48.5 (13.5)
Weight, kg	1094	129 (24.6)
BMI, kg/m^2^	1094	46.6 (7.7)
Sex, female	1093	819 (74.9%)
Ethnicity, white	684	679 (99.3)
Hypertension	1094	403 (36.8%)
Ischaemic heart disease	1094	66 (6.0%)
High Risk of Type 2 diabetes (Pre‐diabetes)	1094	163 (14.9%)
Type 2 Diabetes (T2D)	1094	313 (28.6%)
Depression	1094	516 (47.2%)
Obstructive Sleep apnoea (OSA)	1094	187 (17.1%)
Osteoarthritis	1094	368 (33.6%)
Abnormal liver function	1094	297 (27.1%)
Learning Disability	1094	8 (0.73%)
Edmonton Obesity Scoring System (EOSS) score	1090	
EOSS score, 0 points		85 (7.8%)
EOSS score, 1 point		388 (35.6%)
EOSS score, 2 points		377 (34.6%)
EOSS score, 3 points		238 (21.8%)
EOSS score, 4 points		2 (0.18%)
Physical Activity GPPAQ	1076	
Physical activity, inactive		642 (59.7%)
Physical activity, moderately inactive		201 (18.7%)
Physical activity, moderately active		134 (12.5%)
Physical activity, active		99 (9.2%)
2 item fruit and vegetable score‐per day	1075	4.0 (2.4)
Obesity Class^a^	1094	
Obesity, class 1		27 (2.5%)
Obesity, class 2		156 (14.3%)
Obesity, class 3		911 (83.3%)

*Note:* Values are mean (SD) or *n* = (%) where indicated.

^a^ Obesity classifications as per NICE guidance https://www.nice.org.uk/guidance/cg189/ifp/chapter/Obesity‐and‐being‐overweight.

Fifty seven percent of FWMS participants had an EOSS score ≥ 2, representing long term illness or disability (long‐term illness 20.4% NWCCG, 17.6% England 2020 [38]). Co‐morbidities at referral are reported in Table S4.

New diagnoses at assessment or during the programme included 27 new diagnoses of T2DM, 44 obstructive sleep apnoea (OSA), 8 hypothyroidism, 17 hypercholesterolaemia and 14 hypertension (annual data are shown in Table S5).

Socioeconomic status was assessed using a proxy measure years of education completed. There was a mean of 14.5% (range 22.8%–11.5%) finishing education by 15 years, mean 65% (range 58.5%–76.5%) completing education aged 15–19 years and a mean of 20% (range 18%–23%) completing education aged 19 years or over during the 5‐year period (see Table S6).

Characteristics of completers and non‐completers are described in Table S7. Differences between completers and non‐completers in baseline weight, sex, T2DM, high risk of T2DM and depression were non‐significant. There were statistically significant differences between the groups in age, BMI, EOSS scores and attendance. Non‐completers were younger, with a lower BMI and EOSS score.

The analyses for change in weight at 3, 6, 9, 12 months and 12 months post‐discharge are shown in Table 2. Change in weight was statistically significant for all timepoints in all subgroups (*p* < 0.05, paired sample *t*‐test). Analyses showed that 35.6% (108) of completers had maintained ≥ 10% change in body weight at 12 months post‐discharge (34.3% (120) for all patients with weight recorded). Using BOCF (all patients) there were 11% (120) of patients who maintained ≥ 10% weight loss at 12 months post‐discharge, while for LOCF analysis (all patients) 17.5% (191) maintained ≥ 10% weight loss. Around 50% of patients maintained ≥ 5% change in body weight at 12 months post‐discharge (56.9% (199) for all patients and 58.4% (177) for completers). For the BOCF and LOCF analyses percentages maintaining ≥ 5% weight loss were 18.2% (199) and 37.7% (412), respectively.

**TABLE 2:** Change in weight over 3, 6, 9 and 12 months and 12 months post‐discharge.

Subset	Weight measure	3 months	6 months	9 months	12 months	12 months post discharge
All (1094)	*n*	937	762	670	620	350
All (with a weight recorded at the specified timepoint)	*n*	907	727	593	595	350
	Baseline, kg	129.5 (24.4)	129.9 (25.2)	131.4 (25.1)	130.2 (25.2)	129.9 (26.4)
	Follow‐up, kg	126.1 (24.1)	124.3 (24.9)	124.4 (24.7)	122.0 (24.1)	119.3 (25.6)
	Change, kg	−3.4 (0.2)	−5.6 (0.2)	−7.1 (0.3)	−8.2 (0.3)	−10.6 (0.8)
	Change, %	−2.6 (3.4)	−4.3 (4.6)	−5.3 (5.5)	−6.2 (5.8)	−7.8 (10.2)
	Change, ≥ 5%	244 (26.9%)	320 (44.0%)	318 (53.6%)	357 (60.0%)	199 (56.9%)
	Change, ≥ 10%	36 (4.0%)	88 (12.1%)	117 (19.7%)	144 (24.2%)	120 (34.3%)
Completers^a^	*n*	552	552	527	560	303
	Baseline, kg	130.8 (25.2)	130.6 (25.1)	131.3 (24.9)	130.7 (25.1)	130.9 (26.2)
	Follow‐up, kg	127.0 (24.7)	124.6 (24.6)	123.8 (24.1)	122.4 (24.0)	119.5 (25.1)
	Change, kg	−3.9 (0.2)	−6.0 (0.3)	−7.5 (0.3)	−8.3 (0.3)	−11.4 (0.8)
	Change, %	−2.9 (3.3)	−4.5 (4.5)	−5.6 (5.4)	−6.2 (5.8)	−8.3 (10.3)
	Change, ≥ 5%	165 (29.9%)	259 (46.9%)	297 (56.4%)	336 (60.0%)	177 (58.4%)
	Change, ≥ 10%	26 (4.7%)	72 (13.0%)	112 (21.3%)	133 (23.8%)	108 (35.6%)
Baseline observation carried forward^b^	*n*	1094	1094	1094	1094	1094
	Baseline, kg	129.1 (24.6)	129.1 (24.6)	129.1 (24.6)	129.1 (24.6)	129.1 (24.6)
	Follow‐up, kg	126.3 (24.4)	125.4 (24.5)	125.3 (24.3)	124.6 (24.2)	125.7 (24.7)
	Change, kg	−2.8 (0.1)	−3.7 (0.2)	−3.8 (0.2)	−4.5 (0.2)	−3.4 (0.3)
	Change, %	−2.2 (3.3)	−2.9 (4.2)	−2.9 (4.8)	−3.4 (5.3)	−2.5 (6.8)
	Change, ≥ 5%	244 (22.3%)	320 (29.3%)	318 (29.1%)	357 (32.6%)	199 (18.2%)
	Change, ≥ 10%	36 (3.3%)	88 (8.0%)	117 (10.7%)	144 (13.2%)	120 (11.0%)
Last observation carried forward^b^	*n*	1094	1094	1094	1094	1094
	Baseline, kg	129.1 (24.6)	129.1 (24.6)	129.1 (24.6)	129.1 (24.6)	129.1 (24.6)
	Follow‐up, kg	126.3 (24.4)	125.0 (24.4)	124.3 (24.3)	123.7 (24.2)	123.1 (24.5)
	Change, kg	−2.8 (0.1)	−4.1 (0.2)	−4.8 (0.2)	−5.4 (0.2)	−6.0 (0.3)
	Change, %	−2.2 (3.3)	−3.2 (4.4)	−3.7 (5.0)	−4.1 (5.5)	−4.5 (7.4)
	Change, ≥ 5%	244 (22.3%)	360 (32.9%)	412 (37.7%)	440 (40.2%)	412 (37.7%)
	Change, ≥ 10%	36 (3.3%)	95 (8.7%)	136 (12.4%)	162 (14.8%)	191 (17.5%)

*Note:* Values are mean (SD) for all except ≥ % or ≥ 10% weight change which is *n* = (%).

^a^ A completer is defined as having attended at least nine appointments and having a weight recorded at 12 months. The number of attendances in completers ranged from 9–24.

^b^ Weight was carried forward where missing at an individual timepoint. All *p*‐values were < 0.05 and statistically significant using Bonferroni‐adjusted *p*‐values (calculated separately for each sub‐set).

Of those who attended the service 762 people (69.7%) attended for at least 6 months and 539 (49.3%) attended for 9 or more months defined by having a weight recorded at the 9‐month point. Five hundred and sixty people completed the programme with a final weight at 1 year after referral; some of those people did not have a weight recorded at the actual 9‐month point but did go on to complete the programme. The analyses for secondary outcomes at 3, 6, 9 and 12 months are shown in Table 3. Data at 12 months post‐discharge were not available for these outcomes. Changes in secondary outcomes were statistically significant for all timepoints (*p* < 0.05, paired sample *t*‐test). At 12 months EQ_VAS score had increased by 19.1 (SD 1.2), while fruit and vegetable portion consumption/day had increased by 1.47 (SD 0.12). Physical activity GPPAQ score also increased by 0.54 (SD 0.06), while systolic and diastolic blood pressure decreased by 5.71 mmHg (SD 0.66) and 3.19 mmHg (SD 0.55), respectively. For patients with T2DM HbA1c decreased by 5.7 mmol/mol (SD 1.5) at 12 months.

**TABLE 3:** Change in secondary outcomes over 3, 6, 9 and 12 months.

Measure		3 months	6 months	9 months	12 months
EQ‐VAS (Visual Analogue Scale)	*n*	848	664	513	434
	Baseline	47.7 (22.0)	48.3 (22.4)	47.7 (22.4)	48.0 (22.7)
	Follow‐up	52.7 (21.6)	58.3 (20.8)	62.0 (20.2)	67.0 (20.7)
	Change	5.0 (0.6)	10.0 (0.8)	14.3 (0.9)	19.1 (1.2)
Fruits and vegetables, portions/d	*n*	848	657	508	422
	Baseline	4.04 (2.39)	4.14 (2.40)	4.18 (2.45)	4.25 (2.45)
	Follow‐up	5.04 (1.98)	5.40 (1.88)	5.52 (1.83)	5.72 (1.78)
	Change	1.00 (0.07)	1.26 (0.09)	1.33 (0.11)	1.47 (0.12)
Physical activity, category^a^	*n*	857	667	517	439
	Baseline	1.73 (1.02)	1.68 (0.99)	1.70 (0.99)	1.73 (1.01)
	Follow‐up	1.99 (1.11)	2.15 (1.14)	2.18 (1.17)	2.27 (1.20)
	Change	0.27 (0.03)	0.47 (0.04)	0.49 (0.05)	0.54 (0.06)
Systolic blood pressure, mm Hg (checked at baseline and final visit)	*n*	—	—	—	378
	Baseline	—	—	—	127.89 (13.00)
	Follow‐up	—	—	—	122.19 (10.34)
	Change	—	—	—	−5.71 (0.66)
Diastolic blood pressure, mm Hg (checked at baseline and final visit)	*n*	—	—	—	378
	Baseline	—	—	—	74.10 (9.67)
	Follow‐up	—	—	—	70.90 (8.65)
	Change	—	—	—	−3.19 (0.55)
HbA1c, mmol/mol (only checked at 12 months in participants with type 2 diabetes)	*n*	122	131	95	102
	Baseline	57.5 (16.8)	56.7 (16.0)	58.1 (17.1)	58.8 (18.3)
	Follow‐up	54.0 (14.2)	53.3 (15.8)	53.8 (16.5)	53.0 (14.7)
	Change	−3.4 (1.1)	−3.5 (1.1)	−4.3 (1.6)	−5.7 (1.5)

*Note:* Values are mean (SD). Table shows results from all available data in whole cohort. Change in outcomes were statistically significant for all timepoints (*p* < 0.05, paired sample *t*‐test) and statistically significant using Bonferroni‐adjusted *p*‐values (calculated separately for each outcome).

^a^ Physical activity was defined by GPPAQ in four categories where 1 = least active and 4 = most active.

Predictors of weight loss are shown in Table 4 and were older age, higher weight, BMI and high risk of diabetes (model 1). Predictors of weight maintenance at 12 months post‐discharge were older age, higher weight, BMI and a higher number of attendances (model 1). At 12 months post discharge, few participants had undergone bariatric surgery, although some were awaiting this intervention. GLP‐1 agonist obesity management medications were not available to NHS patients during the evaluation period.

**TABLE 4:** Predictors of weight change.

	Weight change 12‐months^a^						Weight change 12‐months post‐discharge^a^					
	Model 1			Model 2			Model 1			Model 2		
	*n*	Change, kg	*p*	*n*	Change, kg	*p*	*n*	Change, kg	*p*	*n*	Change, kg	*p*
Age (per year)	513	−0.07 (0.03)	< 0.01	513	−0.14 (0.03)	< 0.01*	289	−0.17 (0.06)	< 0.01*	289	−0.27 (0.07)	< 0.01*
Baseline weight (per kg)	513	−0.10 (0.01)	< 0.01*	513	−0.16 (0.03)	< 0.01*	289	−0.17 (0.03)	< 0.01*	289	−0.19 (0.07)	< 0.01
Baseline BMI (per kg/m^2^)	513	−0.25 (0.04)	< 0.01*	513	0.13 (0.09)	0.13	289	−0.58 (0.10)	< 0.01*	289	−0.12 (0.22)	0.60
EOSS score (per point)	513	−0.48 (0.43)	0.26	513	−0.09 (0.47)	0.86	289	−1.69 (1.00)	0.09	289	−0.12 (1.11)	0.91
Attendances, number	513	−0.09 (0.08)	0.27	513	−0.09 (0.08)	0.26	289	−0.48 (0.18)	< 0.01	289	−0.38 (0.17)	0.03
Sex (male vs. female)	513	−1.53 (0.85)	0.07	513	2.24 (1.08)	0.04	289	0.53 (2.03)	0.79	289	5.17 (2.63)	0.05
High Risk of Type 2 diabetes (Pre‐diabetes) (yes vs. no)	513	−3.37 (1.04)	< 0.01*	513	−1.99 (1.02)	0.05	289	−3.31 (2.47)	0.18	289	−1.78 (2.37)	0.45
Diabetes (yes vs. no)	513	0.51 (0.78)	0.52	513	0.63 (0.84)	0.45	289	−1.14 (1.85)	0.54	289	−0.89 (1.99)	0.65
Depression (yes vs. no)	513	1.55 (0.73)	0.03	513	1.63 (0.70)	0.02	289	−1.20 (1.75)	0.49	289	0.15 (1.66)	0.93

*Note:* Regression analyses‐ change in weight in kg as the dependent variable and the relevant predictors as the independent variable, model 1 each predictor was modelled individually, model 2 all predictors were entered into the same model.

^a^ Including 2 item fruit and vegetable score and GPPAQ measure of physical activity in both models did not change the statistical significance of the result and reduced the number of data points included.

* Indicates statistical significance using Bonferroni‐adjusted *p*‐value (calculated separately for each model).

Although exact data were not recorded, it was estimated that under 10 people a year were prescribed Orlistat and under 20 people a year used a TDR (C Hughes personal communication). In addition, many FWMS patients were interested in bariatric surgery and around 30–50 people a year were assessed as appropriate and prepared for surgical referral (C Hughes personal communication).

## 4 Discussion

This service evaluation confirms that it is possible to deliver effective multidisciplinary specialist weight management services in primary care. The weight loss results were consistent, despite the complexity of people attending the service and were in line with results from other UK based SWMS [22, 23, 39, 40]. Mean BMI was 46.6 kg/m^2^ compared with 44.1 kg/m^2^ in the earlier FWMS evaluation reported by Jennings in 2014 [20]. In this group of medically complex patients, all of whom had previously engaged with other weight loss interventions, the results were encouraging with weight loss of ≥ 5% in 60% of patients (72.6% in FWMS cohort 2014), ≥ 10% in 23.8% of patients (27% FWMS cohort 2014) and 6.2% mean weight loss in completers (8% in FWMS cohort 2014). The ambitious KPI of > 5% weight loss in 70% of completers was not achieved, possibly reflecting the high BMI and complexity of the patients. But the 5% weight loss results are at the top end of the range described by other similar SWMS [39, 40]. All weight changes were statistically significant, as were improvements in diet, physical activity, quality of life, blood pressure and HbA1c (in people living with T2DM). Twelve months post‐discharge mean weight loss was 8.3% (SD 10.3), *n* = 350 in completers using all available data. Follow‐up data 12 months post‐discharge, although incomplete, is encouraging, with maintenance of some weight loss and BOCF mean weight loss in the whole cohort was 2.5% (SD 6.8), *n* = 1094 at 12 months post‐discharge.

Most patients at FWMS had a BMI indicating class 3 obesity or above and were medically complex (EOSS score ≥ 2 in 57%); many had previously tried multiple weight loss interventions, so the weight loss results should be compared to similar patient cohorts. Some extremely complex patients, such as those on renal dialysis, were excluded at referral and referred to secondary care SWMS (Figure 1). The FWMS BMI range was similar to the Aintree Loss community‐based SWMS [39]. However, a larger proportion (60%) had categorical weight loss of > 5% at FWMS compared with Aintree Loss (24.1%). This may partially be explained by differences in attendance rates. Sixty‐nine‐point 7% of people attended FWMS for ≥ 6 months, whilst at the Aintree Loss service, 33.6% attended for over 6 months [39]. The Aintree loss service offered appointments for up to 2 years, the participants included 58.9% of people living in the most deprived decile of the Multiple deprivation index and the paper reported data from 2009–2013 preceding FWMS data [39] precluding direct comparisons. The Glasgow and Clyde Weight Management Service [40] which was delivered at community sites, with similar inclusion criteria to FWMS but a slightly lower BMI range attending, used a different definition of completers, showing that 44% of completers (*N* = 310) lost 5% or more of weight in 6 months. One thousand eight hundred and thirty‐eight Glasgow and Clyde patients had a first clinic visit during this period (October 2008 to September 2009).

The FWMS drop‐out rate may reflect the monthly visits being too far apart to achieve engagement, travel problems (single site accepting people from a wide geographical radius), limited pharmacotherapy and waiting time for bariatric surgery.

People over 65 years of age were under‐represented at FWMS in comparison with Norfolk or England populations [38]. This might relate to referral rates, perception of the service by people living with obesity (PLwO) or problems with distance to the service. There were statistically significant differences between completers and non‐completers. Non‐completers were younger, with a lower BMI and EOSS score. Further work to understand how to make the service more attractive to younger people with lower classes of obesity would be useful; for example, increased digital access might be attractive to this group [22]. After 2019, FWMS developed more group‐based interventions and used group consultations both in person and online successfully. During COVID‐19, FWMS pivoted to telephone‐based and online consultations and these continued in part long‐term, which may have improved the accessibility of the service [22]. These adaptations could lower costs due to reduced needs for physical consultation rooms and staff costs.

A systematic review of SWMS in the UK by Alkharaiji showed a short to medium positive effect on weight (this review included the previous data from FWMS published in Jennings 2014) [19, 20]. However, it only found limited long‐term data for SWMS, which were from a heterogenous group of SWMS (90% of services provided no data at 18 and 24 months after recruitment) [19, 20]. In this review, 43% of patients had 5% categorical weight loss at 12 months (seven studies) (FWMS 2014 data was around 60% of patients) [19, 20]. The only study included in this review which provided 5% weight loss data at 18 and 24 months was the previous FWMS study by Jennings (47.9% of patients and 44.4% of patients at 18 and 24 months respectively) [19, 20]. In comparison, in this current evaluation, 56.9% of patients achieved > 5% weight loss at 24 months (using all available data) (Table 3). The significant improvements in physical activity we have found in this FWMS evaluation contrast with the findings of the systematic review by Alkharaiji, where an initial improvement in physical activity was not maintained [19]. Additionally, completers in this evaluation had reductions in blood pressure and HbA1c (in people living with T2DM) equivalent to that achieved by adding in an additional medication for either condition [41].

Since the inception of the FWMS in 2009 there has been increasing evidence to show the effectiveness of TDR [34, 35]. FWMS included TDR interventions as the evidence emerged. The Doctor Referral of Overweight People for Low Energy Treatment (DROPLET) trial involved NHS GPs randomly allocating 278 PLwO to usual care or to a Cambridge Weight Plan consultant for an 810 kcal/d weight loss programme using TDR, followed by maintenance for 1 year [34]. This trial was conducted in primary care and any medication changes were managed by the participant's own GP. The results in the TDR group demonstrated a mean weight change of −10.7 kg (SD 9.6) (control −3.1 kg (SD 7.0)), with > 5% weight loss in 73% of participants and > 10% in 45% of participants. DROPLET trial participants were less medically complex and had a lower mean BMI of around 37 kg/m^2^ compared with FWMS mean BMI 47 kg/m^2^. TDR may have been underutilised at FWMS. However, FWMS patients reported that the cost of products was a barrier even with an NHS discount and that the solely milk‐based diet replacement option was not very palatable. There was good communication and shared care protocols with local bariatric centres, but the requirement for FWMS to refer the participant back to their GP for onward surgical referral introduced delay to the system, which was distressing for patients. Pharmacotherapy on the NHS was limited to Orlistat during the study period. The approval of new obesity management medications (OMMs) such as GLP‐1 agonists for use within the NHS is likely to improve weight loss and obesity related co‐morbidities [16, 24, 42, 43]. Seventy‐one of the patients in this evaluation would have met NICE criteria for Liraglutide (6.9%) and 565 (52%) would have met NICE criteria for Semaglutide [42, 43]. Even more PLwO might be eligible for Tirzepatide which has recently been approved for use outside hospital settings [25]. Although not all those eligible would have chosen to accept pharmacotherapy, some may have had contraindications that would prevent the use of these OMMs. The roll‐out of Tirzepatide to primary care in the UK may reduce the need for SWMS, since behavioural management support in addition to OMMs may suffice for many patients [24]. However, there will remain some complex patients who require additional specialist medical, psychological, dietetic or physiotherapy input and FWMS shows that this can be provided by a primary care SWMS.

Recruitment to FWMS and service development was significantly impaired by short‐term commissioning. After the initial 3‐year contract, there were multiple 1‐year extensions. This was confusing for both referring GPs and patients since recruiting was paused several times whilst waiting to have continued funding confirmed. This affected referral rates (Table S3), service development and staff morale and retention.

It is important to note that FWMS provided additional value beyond clinical management, which included delivering educational events with an emphasis on reducing weight stigma, how to start a conversation about weight, service delivery and encouraging shadowing by visiting healthcare professionals. These educational activities had the potential to help increase the frequency of discussions about weight in primary care and subsequent referrals to a SWMS. The ACTION‐IO study identified a delay in HCP raising the topic of weight and referring PLwO to appropriate services, with a mean delay of 9 years in the UK between PLwO starting to struggle with their weight and discussing it with an HCP [44]. Lack of training in discussing and assessing obesity was a barrier for HCPs and FWMS educational activities may have helped address this [44]. FWMS patients and staff engaged in numerous research projects and clinicians presented at academic conferences [45]. FWMS staff also had significant roles in developing protocols nationally on managing post‐bariatric surgery patients in primary care within the British Obesity and Metabolic Surgery Society GP Hub (https://bomss.org/gp‐hub/).

This evaluation has presented data from the pre‐COVID‐19 era. During the COVID‐19 pandemic there was a cessation of new referrals. FWMS pivoted to online and telephone consultations to support those in the service and developed online group consultations for eating behaviour support such as emotional eating. FWMS also developed their website, including a members‐only area containing the workbook material. FWMS re‐introduced the offer of face‐to‐face consultations as soon as the advice allowed and continued a mixed model of face‐to‐face and remote consultations.

This is an evaluation of one of the few examples of a primary care‐based SWMS. There is evidence supporting the cost‐effectiveness of weight management in primary care in the UK using the Counterweight behavioural programme, but that did not involve a full multidisciplinary team or such complex patients [21]. There is increasing evidence about the high levels of health and social care utilisation by people with severe obesity and medication costs increase both with obesity and obesity‐related co‐morbidities [3, 21]. Engaging with PLwO earlier in their weight trajectory should reduce both co‐morbidities and health and social care costs. A local primary care‐based service might be a more accessible and acceptable option to patients to help achieve this. A recent study explored the community health and long‐term care needs of people with severe obesity, including those who are housebound [3]. Total annual service costs incurred by participants varied from £2053 to £82 792 (mean £26 594) and total mean annual costs increased by ascending BMI category, up to BMI 70 kg/m^2^ [3]. Primary care has responsibility for providing medical care for housebound patients. However, FWMS was not funded to provide care for housebound people and this omission should be rectified by commissioners in future.

The FWMS service was commissioned for £250 000 for 200 completers per annum (2010). This gives a cost of £1250 per completer. This did not include the cost of drugs, which were prescribed by the participants' own GP. The annual budget did not include the initial cost of developing the service. Set up costs, including equipment and staff training, were originally funded by an NHS East of England Innovation and Enterprise award of £237 000. Addition donations for equipment were made by local retailers. FWMS worked closely with Public Health and local providers to efficiently utilise pre‐existing resources such as physical activity support and cookery demonstrations. The service ceased in 2022 when a whole Norfolk Tier 3 SWMS was commissioned.

### 4.1 Strengths and Limitations

Strengths include a detailed demographic description of the 1094 patients and relevant weight and secondary outcome measures. The one‐year post‐programme follow‐up data adds longer term information on weight outcomes, which is lacking in the literature [19].

This evaluation provides real world data from primary care on managing the important disease of obesity. The majority of PLwO present and are managed in primary care in the UK and recent NICE guidance supports the expansion of obesity management in primary care [16, 24].

Limitations included that since this was a service evaluation it used only routinely available data and there was no funding to collect further data or opportunity to collect data such as reasons for people declining to join the service or dropping out. There was a brief technical software problem, which was identified and corrected. It is possible that a small number of data points remained missing.

FWMS was a complex intervention; it was difficult to extract all the data from clinical systems and analyse individual components. There was a varied number of attendances in completers (ranged from 9–24) and we did not record which type of HCP conducted each appointment or whether the appointment was group or individual. Since the number of appointments varied, it was not possible to make direct comparisons with other similar services that offered a different number of appointments [19, 39, 40]. The cohort of patients included those on TMR, Orlistat and preparing for bariatric surgery, but it was not possible to analyse those interventions separately. The data were collected prior to the availability of newer OMMs in the NHS.

When collecting one‐year post‐discharge weight data, a potential bias was introduced as PLwO may be more likely to respond to requests for follow‐up data if they have maintained or lost further weight. A few of the follow‐up weights were self‐reported, which might reduce accuracy. In the future, the National Obesity Audit [46] may collect more data from community or primary care‐based services, but during this evaluation period these data were not centrally collated. Since obesity is a chronic condition, collecting longer‐term follow‐up data would be valuable beyond the one‐year point.

The generalisability of the data is limited by subsequent developments such as an increase in online consultations and interventions; however, changing the method of delivery would be possible as demonstrated by the changes made at FWMS during the COVID‐19 pandemic.

### 4.2 Implications for Policy

The future need for UK primary care‐based specialist weight management services has yet to be quantified, as OMMs are being rolled out in primary care [24] with behavioural support (often online). In the UK, recent guidance from NICE supports the transfer of care for PLwO to primary care where possible, including the prescribing of new OMMs where clinically appropriate, but is limited by funding and commissioning and being rolled out over a prolonged period [16, 24, 25, 47]. A detailed mapping exercise of four hospital‐based SWMS (three in England and one in Wales) by Zhang and colleagues [48] demonstrated variations in entry criteria, programme content, staff mix and method of delivery. It also revealed resource constraints, with long waiting lists and problems with communication, for example, between primary and secondary care. They propose a model of care with community‐based services (tier 2), supported by a primary care physician or nurse specialist (Tier 2.5) who could prescribe OMMs and the most complex patients to attend SWMS (Tier 3), which could be based in primary or secondary care. Patients could be stepped up or down as appropriate within these layers of service. This evaluation suggests that if staffed and resourced appropriately, much of the SWMS could safely and effectively be provided in primary care. However, all parts of the pathway would need to be commissioned and resourced. The UK government's policy paper, 10 Year Health Plan for England [47] together with NHS England's plans to establish Neighbourhood Health Centres [49], are likely to lead to more care being delivered in the community rather than secondary care. There is also increased interest in developing new models of care specifically for obesity, for example, the recent £85 million funding call, Obesity Pathway Innovation Programme [50]. Therefore, this evaluation of a model for a specialist obesity service delivered within primary care will likely be of significant interest to those involved in health service design and policy [47, 49, 50].

## 5 Conclusion

There is a lack of evidence on long‐term non‐surgical interventions for people with severe obesity including long‐term weight data [23]. This paper reports data from an NHS primary care‐based SWMS, treating medically complex patients, including data at 12 months post‐programme end. The evaluation period was pre‐COVID‐19 and before the introduction of the new GLP‐1 agonist OMMs. The results of this evaluation, which included data from 1094 patients, show consistency in weight loss achieved over the 5‐year period and clinically and statistically significant improvements in diet, physical activity, quality of life, blood pressure and HbA1c. Importantly, a positive effect on weight maintenance 1 year after discharge was demonstrated even using BOCF.

There is potential to increase the effectiveness of SWMS services through the introduction of the newer OMM (over half of the patients attending FWMS would have been eligible for a GLP‐1 agonist using NICE criteria). Further improvements might also be achieved by increasing the use of total diet replacements [11, 34, 35] and/or by increasing the use of remote support for weight maintenance after discharge [51, 52].

The approval of Tirzepatide for the management of obesity outside of hospital settings offers an opportunity to upskill and increase the involvement of primary care HCPs in obesity management [24]. However, there will still be a cohort of complex patients who require more intensive, specialist medical, dietetic and psychological interventions [48]. A primary care‐based SWMS could act as a supportive hub for complex patients and this evaluation suggests that a primary care‐based SWMS is feasible, would fit within proposed NHS pathways [7, 47, 49, 50] and would be increasingly effective with the inclusion of the newer OMMs.

## Author Contributions

C.A.H. checked and cleaned the data, A.J. did the statistical analysis, H.M.P., S.E. and C.A.H. wrote the paper with expert input from N.S. and A.J. All authors were involved in writing the paper and had final approval of the submitted and published versions.

## Funding

The authors have nothing to report.

## Conflicts of Interest

H.M.P. is a BOMSS council member and an advisory panel member for the UK Coalition for People Living with Obesity. She has received honoraria for educational events and clinical pathway development consultancy from Johnson & Johnson, Novo Nordisk, Boston Scientific, AbbVie and Radcliffe Group. H.M.P. was a member of the NICE obesity clinical guidelines NG246 committee and NICE obesity quality standards for obesity QS212 committee. C.A.H. has received honoraria for educational events from NovoNordisk, Ethicon and Menwell health. S.E. is an NIHR clinical lecturer. The remaining authors declare no conflicts of interest.

## Supporting information


**Table S1:** Key Performance Indicators for FWMS‐Quality Quarterly reporting (abridged for brevity).
**Table S2:** Referrals to FWMS (1st April 2014 to 28th March 2019).
**Table S3:** Referrals per year (from annual report data).
**Table S4:** Co‐morbidities at referral from annual report data.
**Table S5:** New Diagnoses detected from annual report data.
**Table S6:** Socio‐economic status (years of education) from annual Report data.
**Table S7:** Characteristics of non‐completers and completers.

## Data Availability

The data that support the findings of this study are available from the corresponding author upon reasonable request.
