# Supplementary material for: A Service Evaluation of a Specialist Multi‐Disciplinary Weight Management Service Based in Primary Care Including Long‐Term Follow‐Up Data
Source: Clin Obes. 2026 Jul 2;16(4):e70096. doi: 10.1111/cob.70096 (PMC13324968; doi:10.1111/cob.70096)
Supplement: Supplementary file 1 — Table S1: Key Performance Indicators for FWMS‐Quality Quarterly reporting (abridged for brevity). Table S2: Referrals to FWMS (1st April 2014 to 28th March 2019). Table S3: Referrals per year (from annual report data). Table S4: Co‐morbidities at referral from annual report data. Table S5: New Diagnoses detected from annual report data. Table S6: Socio‐economic status (years of education) from annual Report data. Table S7: Characteristics of non‐completers and completers. [file COB-16-e70096-s001.pdf]

A service evaluation of a specialist multi-disciplinary weight management service based in primary care including long-term follow-up data

Parretti Helen M[1] Hughes Carly A[1,2], Erskine Sally[1], Steel Nicholas[1,] Jennings Amy[1,3]

1. Norwich Medical School, University of East Anglia, Norwich Research Park, Norwich, NR4 7TJ
2. Fakenham Medical Practice, Meditrinia House, Trinity Road, Fakenham, Norfolk, NR21 8SY
3. School of Biological Sciences, Queens University Belfast, University Road, Belfast, Northern Ireland, BT7 1NN

Keywords: obesity, primary care, general practice, specialist weight management service.

Corresponding author: Dr Carly Anna Hughes Fakenham Medical Practice, Meditrinia House, Trinity Road, Fakenham, Norfolk, NR21 8SY. cah216@cam.ac.uk

Supplementary Information

Commissioning Information

The service was initially commissioned by North Norfolk Clinical Commissioning group (NNCCG), but both South Norfolk Clinical Commissioning group (SNCCG) and West Norfolk Clinical Commissioning (WNCCG) then purchased places on the service. A few people were referred from the Yarmouth and Waveney Clinical commissioning group (YWCG) on an individual funding basis (IFR) from other CCGs.

Table S1 Key Performance Indicators for FWMS-Quality Quarterly reporting (abridged for brevity)

Reference	Requirement	Method of Measurement
1	% of service completers 'satisfied' or 'very satisfied'	% of service completers *in the previous quarter to be 'satisfied' or 'very satisfied'
2	% of service users who have gained body weight 6 months after treatment starts	% of service completers in the previous quarter that have gained weight from their original body weight
3	% of service completers who have maintained original body weight 12 months after treatment starts	% of service completers in the previous quarter maintaining original body weight
4	% of service completers who have lost 1-5% total body weight 6 months after treatment starts	% of service completers in the previous quarter achieving 1-5% body weight loss
5	% service completers who have lost >5% total body weight 12 months after treatment starts	% of service completers in the previous quarter achieving 5.1-10% body weight loss
6	% of service completers who have lost >10% total body weight 12 months after treatment starts	% of service completers in the previous quarter achieving more than 10% body weight loss
7	% of patients who have increased physical activity	% of service completers in the previous quarter with an increase in physical activity score at 6 months, or if already active, those who maintain a high activity

Reference	Requirement	Method of Measurement
	levels 6 months after treatment starts	
8	% of patients who have increased physical activity levels 12 months after treatment starts	% of service completers in the previous quarter with an increase in physical activity score at 12 months, or if already active t, those who maintain a high activity
9	Patients will receive comprehensive assessment in line with NICE guidance	Audit by CCG
10	Patients not suitable for the programme being referred to GP with feedback/ recommendations	% of patients considered inappropriate for the service and referred back to GP
11	Mean weight loss and loss post engagement with the service at 12 months	Number of completers weight/weight loss 12 months after discharge from programme.
12	Percentage of service completers in Tier 3 with an improvement in EQ-5D score on completion	Number of service completers in the previous quarter with an improvement in EQ-5D -5L QoL score

** A completer is defined as having attended at least 9 appointments and having a weight recorded at 12 months.*

Additional Contract specified outcomes:

- A reduction in patients' weight of ≥1-5% within 6 months of joining the programme in 50% of attenders
- A reduction in patients' weight of ≥5% within 12 months of joining the programme in 70% of attenders
- A reduction in obesity related drug prescriptions (CCG audit)
- An improvement in QoL scores using EuroQol EQ 5D 5L VAS
- An increase in physical activity using General Practice Physical Activity Questionnaire (GPPAQ)
- An increase in healthy eating as measured by 2 item food frequency score (2 item FFS) fruit and vegetables

Staff Training

All HCPs had appropriate clinical training and World Obesity SCOPE accreditation. In addition, the doctors and nurses attended multiple obesity related courses, sat in on clinics in secondary care, both bariatric surgery and bariatric physician clinics, and all HCPs attended training on dealing with disordered eating. There were regular inhouse training sessions, and a clinical core group including clinical psychologist, endocrinologist, eating disorders specialist and patient

representatives reviewed protocols and advised where appropriate. There were joint protocols for managing LELD, and preparing patients for bariatric surgery co-produced with the Norfolk and Norwich University Trust Hospital and the Luton and Dunstable Bariatric unit.
FWMS was research active and core staff had research training including Good Clinical Practice training. All staff underwent annual appraisals and were encouraged to have educational development plans. CH developed education e-learning modules for the RCGP and other providers and lectured nationally and internationally.

FWMS pathway
After medical assessment, examination, and discussion on the health risks of obesity and benefits of weight loss, an initial 5% weight loss goal was agreed. Participants were encouraged to set individual SMART goals and supported to achieve them. Each participant was also given a bespoke FWMS workbook with details of the programme, diet, physical activity, sleep, eating behaviour advice and a dietary diary. The core programme was delivered by health trainer equivalents, obesity specialist nurses, advanced nurse practitioners and dietitians. Every participant was initially discussed at the weekly multidisciplinary team meeting (MDT), and clinical decisions were made, including internal referrals to the emotional eating group, bariatric surgery information group, dietitian, psychological therapist or medical exercise specialist (Figure 1). All Healthcare Professionals (HCPs) within the MDT brought back queries on individual participants to MDT as required at any stage (Figure 2). Once enrolled, participants attended regular monthly individual consultations, behavioural changes were discussed and individualised goals were agreed including physical activity, sleep, dietary and stress management behavioural changes. At subsequent appointments weight was measured, and diet and behaviour change goals were mutually agreed. Once the initial 5% target was achieved further progressive 5% weight loss targets were set. In addition, some participants attended supervised sessions at the on-site gym (with specialist bariatric gym equipment), or psychological interventions either individually or in groups. Telephone appointments were occasionally used, but most contacts were face-to-face at Fakenham Medical Practice.

Table S2 Referrals to FWMS (1st April 2014 to 28th March 2019)

**Total Number of PLwO referred during this period**	**2010**
Referred for post bariatric surgery follow up	17 (not included in analysis)
Referral declined by FWMS	335 (did not meet criteria, or significantly medically complex e.g. renal dialysis, undergoing cancer treatment or requiring specialist secondary care SWMS)
Appointment declined by participant	333
Assessed but not recruited	208 (participant attended initial assessment appointment but declined to enter programme, or assessed by clinician and referred to other service e.g. Eating Disorders Service/secondary care-based SWMS)
Recruited on to programme but first appointment after 28 Mar 2019	23 (not included in analysis)
Recruited on to programme	1094

Annual reports were provided to the commissioners but note that the results below show the number of PLwO completing the programme within a specific financial year. As recruitment was continuous the number of completers partly reflected those referred during the previous financial year and is not synonymous with the data analysed for this paper. Data was locked after the service was decommissioned, so there was no access to the annual report raw data.

Table S3 Referrals per year (from annual report data)

**Referred from 1 April**	**2015**	**2016**	**2017**	**2018**	**2019**
Total referred	428	351	341*	435	455
Referral rejected (not meeting criteria or very complex)	32	35	36	19	32
Did not wish to attend	18	54	107	81	73
Assessed not recruited	23	36	58	45	50
Accepted on to programme	355	226	140*	290	300
Completers	138	141	154	165	169
Drop out	81	51	63	71	80

* The uncertainty of the continuation and funding of the programme in 2017-2018 resulted in a pause in accepting West and South Norfolk Clinical Commissioning Groups referrals from January to March 2018, followed by a large backlog of referrals which were then accepted in March 2018. This skews the figures and many of those referred in financial year 2017/18 did not have their first appointment until April/May 2018. This had an impact on available appointments in March, so some people who would have attended for their final appointment that month had to be offered appointments in April 2018.

Table S4 Co-morbidities at referral from annual report data

	2015	2016	2017	2018	2019
Depression	163 (45%)	43%	50%	46%	46%
T2D	100 (28%)	25%	27%	29%	22%
OSA	62 (7%)	14.5%	15%	18%	12%
OA	132 (37%)	28%	33%	30%	23%
Hypertension	137 (36%)	34%	33%	34%	31%
IHD	18 (5%)	5%	7%	5%	4%
Learning disabilities	5 (1.4%)	2 (0.8%)	5 (4%)	1 (0.3 %)	2 (0.6%)

Table S5 New Diagnoses detected from annual report data

	2015	2016	2017	2018	2019
High risk of T2D	5	26	39	60	57
T2D	6	4	6	2	0
OSA	16	4	3	4	2
Hypercholesterolaemia	9	2	4	2	0
Hypothyroid	2	2	3	0	0
Hypertension	9	2	2	1	0

Table S6 Socio-economic status (years of education) from annual Report data

Years of Education	2016	2017	2018	2019	2020
≥15 years	22.8%	1.2%	22%	15.2%	11.5%
15-19 years	59.2%	76.5%	58.5%	65.4%	65.5%
≥19 years	18%	22.3%	19.5%	19.4%	23%

Table S7 Characteristics of non-completers and completers

**Characteristic**	**Non-completer**		**Completer**		**P=**
	n=	Mean (SD) or n= (%)	n=	Mean (SD) or n= (%)	
Age, baseline years	533	46.9 (13.5)	559	50.1 (13.4)	<0.01
Weight, baseline kg	535	127.5 (23.9)	559	130.7 (25.2)	0.03
BMI, baseline kg/m^2	535	45.8 (7.5)	559	47.3 (7.9)	<0.01
EOSS score, points	531	1.6 (0.9)	559	1.8 (0.9)	<0.01
Attendances, number	334	7.5 (3.6)	508	16.8 (4.4)	<0.01
Sex, male	535	140 (26.2 %)	558	134 (24.0 %)	0.41
High risk of type 2 diabetes (Pre-diabetes)	535	83 (15.5 %)	559	80 (14.3 %)	0.58
Type 2 Diabetes	535	145 (27.1 %)	559	168 (30.1 %)	0.28
Depression	535	257 (48.0 %)	559	259 (46.3 %)	0.57

Values are mean (SD) or n= (%) where indicated. Differences between groups were calculated using independent sample t-tests (for continuous data) or chi-square tests (for categorical data). A completer is defined as having attended at least 9 appointments and having a weight recorded at 12 months.
